# Misdiagnosis of a Giant Uterine Leiomyosarcoma: Clinic and Image Challenges

**DOI:** 10.1155/2017/3568328

**Published:** 2017-07-18

**Authors:** Jila Agah, Sedighe Karimzadeh, Fateme Moharrer Ahmadi

**Affiliations:** ^1^Department of Obstetrics and Gynecology, Faculty of Medicine, Sabzevar University of Medical Sciences, Sabzevar, Iran; ^2^School of Medicine, Sabzevar University of Medical Sciences, Sabzevar, Iran

## Abstract

A 41-year-old woman (G_3_P_2_L_2_Ab_1_) was referred to gynecology clinic with chief complaints of abdominal distension and localized abdominal wall pruritus for three months. She was misdiagnosed with gastrointestinal disorder and ultimately had undergone imaging. Ultrasonography and computed tomography (CT) scan disclosed a huge solid-cystic mass originating from the ovary. On clinical examination the patient had no pain or tenderness and no gynecologic complaints. Laboratory tests showed normal tumor markers and hemoglobin at 8 g/dl. Laparotomy was carried out as diagnosis of ovarian serous cyst adenoma, but a huge tumor with attachment to uterus and ovaries and extension to pelvic floor, peripheral tissues of ureter, and upper abdomen was found. Hysterectomy with bilateral salpingooophorectomy was done. Pathology report demonstrated uterine leiomyosarcoma measuring 40 centimeters and weighing 10 kilograms. In conclusion, as pelvic masses even in a large size may present unspecific symptoms misdiagnosis may occur which lead to overgrowth, local invasion, or other complications. So, it is rather to suggest ultrasonography in patients with persistent abdominal or pelvic symptoms and if needed, more exact diagnostic modalities like magnetic resonance imaging (MRI) could be offered to avoid misdiagnosis and mismanagement.

## 1. Introduction

Pelvic masses are common findings in general gynecology [1]. Benign leiomyomas are the most uterine neoplastic mass which manifest clinically in about 30% of women older than 35 years [2]. Uterine sarcomas include 8% of all uterine malignancies with an incidence of about 0.4 per 100,000 women where leiomyosarcoma (LMS) consists 40% of them [3]. It occurs mostly in the 45–55 years of age. Leiomyosarcomas usually arise de novo from uterine smooth muscles; however rarely they may appear in a preexisting leiomyoma (0.2% of the cases) [4]. The gold standard of treatment for LMS is hysterectomy [5, 6]. Most leiomyosarcomas are accompanied with pain, sensation of pressure, and abnormal uterine bleeding or present only as a rapidly enlarging mass [7]. However, many imaging modalities are available and easy to accelerate the diagnosis; still some gigantic tumors are going to be neglected. Moreover, sometimes these tumors are erroneously reported as ovarian masses on imaging follow-up which can induce severe challenges for surgeon during operation. Herein we represent a huge uterine leiomyosarcoma mismanaged with incorrect diagnosis of dyspepsia for over two months and afterwards operated as misdiagnosis of ovarian mass reported in images.

## 2. Case Presentation

**Figure 1:**
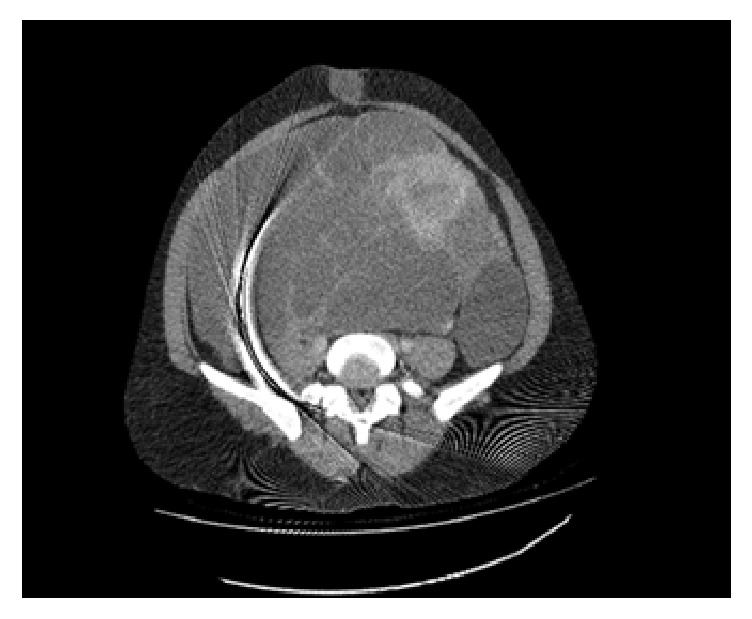
Intravenous contrast CT scan showing a large septated solid-cystic mass with report of serous cyst adenoma.

**Figure 2:**
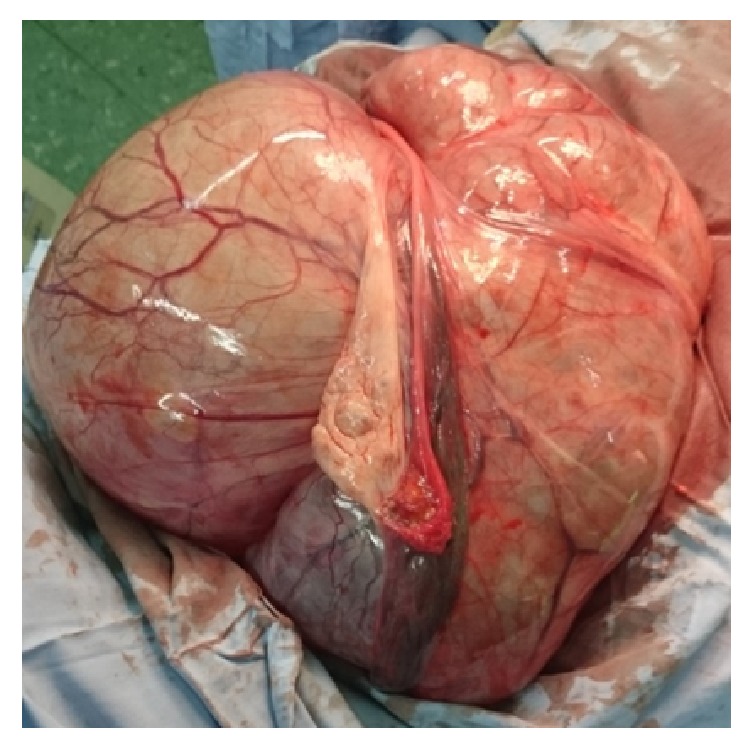
Gross appearance of tumor.

**Figure 3:**
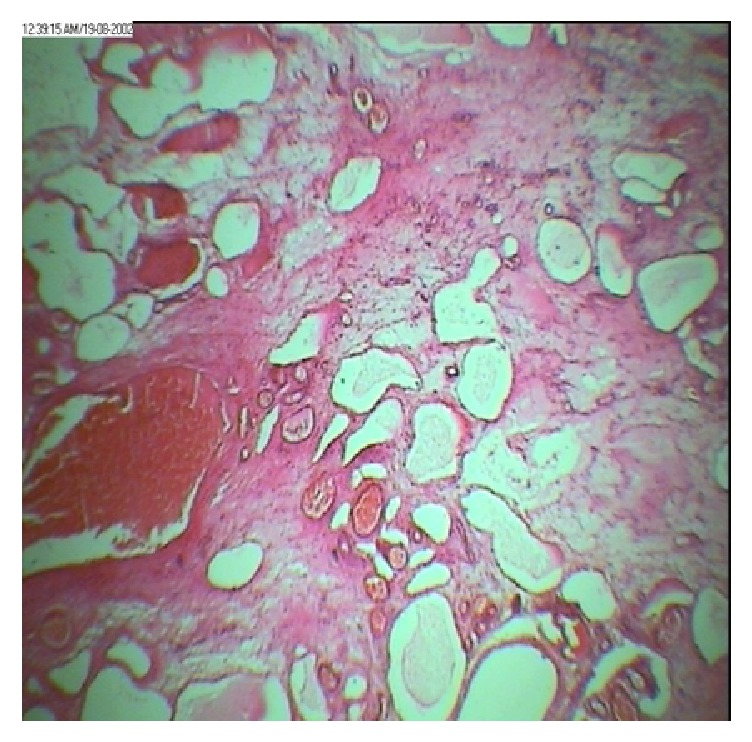
Microscopic appearance of tumor cells.

A 41-year-old woman (G_3_P_2_L_2_Ab_1_) was referred to gynecology clinic with chief complaints of abdominal distension and localized abdominal pruritus for three months. To rule out gastrointestinal disorders, she had visited a general physician given her symptoms. But she found no response to the drugs in spite of a long-term usage and was referred to our clinic after taking ultrasonography. Her past history showed menorrhagia but not menstrual irregularity and dysmenorrhea for several months. Laboratory tests including hemoglobin at 8 g/dl, hematocrit at 28,7%, ferritin at 6 ng/ml, and iron at 29 mcg/dl approved this claim but the patient had not expressed it initially as a complaint. Vital signs were in normal limits. In general appearance, the patient was not cachectic with full activity without vertigo, impairing appetite, constipation, nausea, vomiting, and urinary symptoms, but a distended abdomen that lacks rebound and tenderness. The origin of the mass was not detectable by vaginal examination. Ultrasonography had revealed a huge multicystic septated mass, 40 cm in diameter in abdominal cavity, probably a serous cystadenoma originating from ovary that was extended to epigastric area. Uterine and bladder were reported normal; however, these were impressed by extrinsic pressure of the mass. Uterine observation was not possible with vaginal sonography due to huge ovarian tumor. Tumor markers including cancer antigen-125, carcinoembryonic antigen, *α*FP, and CA19-9 were all within normal limits. Further assessment by computed tomography (CT) scan was done to show the nature of tumor which approved the diagnosis of ovarian serous cyst adenoma (Figure 1); other viscera like kidneys, spleen, and liver were all in normal status; no free fluid and no lymphadenopathy were observed in abdominal cavity. Chest X-ray also did not show any lesion. The patient was hospitalized and advised with operation. The surgery was performed under general anesthesia through a vertical incision. Laparotomy revealed a huge multilocular mass with firm consistency, originating from pelvic with extension to adjacent tissues like ureter and upward to diaphragm with adhesion to ovaries and uterine. Obviously, gentle adhesiolysis to avoid damage to ureter and intestine took a long time. The whole solid-multicystic tumor measuring 40 cm and weighing 10 kg and with attached uterine and ovaries was removed (Figure 2). Lymph nodes and other visceral organs were normal. Regarding previous chronic anemia of the patient, transfusion of two-packed cell-blood was done. Patient's weight was 82 kg before operation and 69 kg subsequently. Three days later, the patient was discharged in a good health status. Pathology report showed uterine leiomyosarcoma (Figure 3). The CT scan for patient's follow-up presents no metastatic mass and lymph node involvement. The patient is under observation and has no problem in one year.

## 3. Discussion

Leiomyosarcomas (LMS) are the most common sarcomatous malignancies of uterus that represent 1-2% of all uterine malignancies [8, 9]. Most patients with leiomyosarcoma have no recognizable risk factors. Patients who carry a germ line p53 gene mutation (Li-Fraumeni syndrome) have an increased risk of soft tissue cancers, including uterine LMS [10]. Some studies have suggested an increased risk for uterine sarcoma among women with obesity and also diabetes [11]. Our case had no identifiable risk factor except her BMI which was 27 kg/m^2^.

The absolute diagnosis of uterine leiomyosarcoma is made by histologic confirmation. In most cases, the diagnosis of these tumors is made by specimen examination in hysterectomized patients who was managed as leiomyoma [5, 12].

The main treatment of LMS is surgical excision which consists of total abdominal hysterectomy and debulking of any tumor invading outside the uterus. It is considered appropriate to preserve ovaries in young women and routine dissection of pelvic and para-aortic lymph nodes, since lymph node involvement is seen in less than 3% of patients [13]. Its five-year survival rate varies from 18.8% to 68% and the risk of recurrence is reported from 45% to 73% [4]. As sarcomas are very aggressive tumors and have the propensity for early hematogenous spread, recurrence and relapse can occur even in completely resected tumors. So it is recommended to prescribe adjuvant chemotherapy in nonmetastatic cases by some authors. The utility of systemic lymph node dissection and adjuvant radiotherapy (RT) in the treatment of uterine LMS remains unclear. Although radiotherapy for reduction of local recurrence and/or chemotherapy for managing distant metastasis have been stated, the literature shows no efficacy concerning overall survival by these modalities actually. Anyway, attention to quality of life which may be influenced by the side effects and toxicity of adjuvant therapy is mandatory [8, 14]. Most cases include women over 40 years old, who are usually referred with abnormal vaginal bleeding (56%), palpable pelvic mass (54%), and pelvic pain (22%) [15]. Kaur et al. reported postmenopausal bleeding and lower abdominal pain in a 60-year-old female with uterine leiomyosarcoma while our patient had no gynecologic problem and no pain [8]. Senol et al. reported a very large leiomyosarcoma in a 62-year-old female presenting with abdominal mass, fatigue, and associated tenderness [16]. Vellanki et al. also reported a uterine leiomyosarcoma in a 40-year-old nulliparous woman presenting with abdominal pain and no menstrual irregularity and no vaginal bleeding [17]; our patient had no vaginal bleeding and no abdominal pain, just complains from 3 months' period of abdominal distension attributed to gastrointestinal diseases. However, abdominal distension is usually attributed to gastrointestinal diseases which leads to misdiagnosis, mismanagement, and overgrowth of tumor [18]. So, it would be reasonable to take an exact history to find out associated symptoms like menorrhagia seen in our case. Besides obtaining a complete history, physical examination, advising appropriate laboratory tests and imaging procedures, is very mandatory particularly in patients who do not respond to usual medications. In this case, severe anemia could be regarded as an alarm sign for her physician if detected. Ultrasonography is considered more appropriate as the first modality for detection of abdominopelvic mass, because it is harmless, available, inexpensive, and applicable by all radiologists and many gynecologists. It cannot reliably determine the origin of huge masses, the nature of malignancy point, and probable invasion of adjacent organs. The role of magnetic resonance imaging (MRI) is developing in the assessment of malignancies. leiomyosarcomas commonly manifest as large infiltrating myometrial mass on MRI with irregular and ill-defined margins and heterogeneous hypointensity on T1-weighted images and also hemorrhage, necrosis, and foci of calcifications may make heterogeneous features of enhancement which can be a hallmark for differentiating from benign leiomyomas [4, 19]. Researchers believe that the sensitivity of MRI for distinguishing the nature and origin of pelvic masses is more than CT scan [4, 20]. To verify, CT scan determined the abovementioned characteristics of the mass in our case but could not define its origin correctly. Subsequently, the patient was operated with diagnosis of ovarian serous cyst adenoma. However, the main advantage of CT scan is that it is cheaper than MRI and more tolerable for patients [4, 20].

In conclusion, pelvic masses even in a large size may be without any specific symptoms. Delay in diagnosis may lead to overgrowth, torsion, or hemorrhagic tear of tumors and invasion to surrounding visceral organs. So, it is considered more appropriate to suggest ultrasonography in patients with persistent abdominal pain or distension who do not respond to recommended drugs and in case of presence of a mass, MRI as a preferable imaging could be offered to avoid misdiagnosis.
